# Tuneable manganese oxide nanoparticle based theranostic agents for potential diagnosis and drug delivery†

**DOI:** 10.1039/d0na00991a

**Published:** 2021-06-07

**Authors:** Kingsley Poon, Zufu Lu, Yves De Deene, Yogambha Ramaswamy, Hala Zreiqat, Gurvinder Singh

**Affiliations:** ARC Centre for Innovative BioEngineering, Tissue Engineering and Biomaterials Research Unit, Sydney Nano Institute, School of Biomedical Engineering, The University of Sydney NSW 2008 Australia hala.zreiqat@sydney.edu.au gurvinder.singh@sydney.edu.au; Department of Engineering, The Biomedical Engineering Laboratory, Macquarie University Sydney 2109 Australia

## Abstract

Among various magnetic nanoparticles, manganese oxide nanoparticles are considered as established *T*_1_ magnetic resonance imaging (MRI) contrast agents for preclinical research. The implications of their degradation properties and use as therapeutic carriers in drug delivery systems have not been explored. In addition, how the chemical composition and size of manganese oxide nanoparticles, as well as the surrounding environment, influence their degradation and MRI contrast properties (*T*_1_*vs. T*_2_) have not been studied in great detail. A fundamental understanding of their characteristic properties, such as degradation, is highly desirable for developing simultaneous diagnosis and therapeutic solutions. Here, we demonstrate how the precursor type and reaction environment affect the size and chemical composition of manganese oxide nanoparticles and evaluate their influence on the nanoparticle degradability and release of the drug l-3,4-dihydroxyphenylalanine (l-dopa). The results show that the degradation rate (and the associated release of drug l-dopa molecules) of manganese oxide nanoparticles depends on their size, composition and the surrounding environment (aqueous or biometric fluid). The dependence of MRI relaxivities of manganese oxide nanoparticles on the size, chemical composition and nanoparticle degradation in water is also established. A preliminary cell viability study reveals the cytocompatible properties of l-dopa functionalized manganese oxide nanoparticles. Overall, this work provides new insights into smartly designed manganese oxide nanoparticles with multitasking capabilities to target bioimaging and therapeutic applications.

## Abstract 2

Here, we demonstrate the design of l-dopamine coated manganese oxide nanoparticle based theranostics for MRI imaging and drug delivery. The release of drug molecules can be controlled by altering the size and chemical composition of nanoparticles.

## Graphical abstract

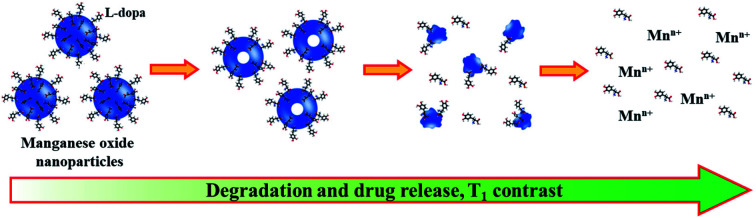

## Introduction

The development of smart and biocompatible magnetic nano-systems armed with multiple capabilities, including targeting, diagnosis *via* magnetic resonance imaging (MRI), and controlled drug delivery without an external stimulus, is the focus of current research in nanomedicine. In the last decade, efforts have been made to design multifunctional superparamagnetic iron oxide nanoparticles (NPs) because of their easy synthesis,^1–3^ rich surface chemistry for functionalization,^4,5^ contrast ability in MRI,^6,7^ and heating efficiency for magnetic hyperthermia.^8,9^ Iron oxide NPs have been favoured because of their unrivalled safety and biocompatibility. Ultrasmall superparamagnetic iron oxide nanoparticles (USPIOs) have been approved by the Food and Drug Administration (FDA) for human diagnosis as a 
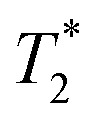
 contrast agent.^10,11^ However, their usage has been limited because the 
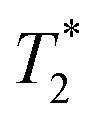
 contrast provides a negative MRI contrast which can be confounded with hypointense signals from bleeding or calcifications.^12^ Furthermore, the advantage of biological storage can lead to retention even months after administration, preluding its continuous use.^13^ Alternatively, gadolinium oxide and iron-doped gadolinium oxide NPs have also emerged as promising candidates due to their high contrast capability.^14–17^ However, renal excretion of these NPs limits their use for clinical applications because the release of Gd ions from NPs can cause nephrogenic systemic fibrosis (NSF).^14^ To use iron oxide and gadolinium oxide NPs as drug delivery vehicles, an additional functionalization strategy, such as the use of stimuli responsive or degradable polymers, is needed to ensure a controlled release of drug molecules.^18–21^ Moreover, the relatively higher stability of these inorganic NPs can cause long-term side effects in an *in vivo* environment. Therefore, the development of degradable and less stable NPs for clinical applications is desirable.

Multifunctional manganese oxide nanoparticles (MONPs) have been developed as prime candidates for clinical applications.^22–26^ Once MONPs are introduced into a biological environment, the oxidation of MONPs produces manganese ions. Ion release is associated with an increase in NMR relaxation rates, generating enhanced MRI signals in *T*_1_ weighted images. These ions can then be readily eliminated by cells *via* the ferroportin protein and from the body *via* fecal-hepatobiliary excretion.^27^ MONPs are also advantageous as they possess two unique properties. First, MONPs offer time-dependent transition in the contrast ability from dark (*T*_2_) to bright (*T*_1_).^28^ This dual contrast mechanism can improve diagnosis accuracy by providing both *T*_1_ and *T*_2_ contrast weighted images. Second, the rate of manganese ion release (the degradation property) is predictable due to the use of MONPs of a predetermined size and concentration, which is a vital characteristic in a biomedical context.^8^ Furthermore, MONPs are antioxidants and help to suppress oxidative stress-related disorders, thus opening avenues beyond diagnostics.^29,30^ Functionalization of MONPs with targeting and drug vectors can transform and enrich them into theranostic NPs.

In the last decade, synthetic techniques have been established to fabricate size- and shape-controlled MONPs.^31–33^ These NPs show unique magnetic properties, *i.e.*, superparamagnetic behaviour at room temperature. These superparamagnetic properties (no magnetization at room temperature) help in preventing the aggregation of these NPs and has encouraged the use of MONPs as MR imaging contrast agents for diagnosis.^34^ However, there are no studies that have explored the degradation properties of MONPs as a premise for predictable drug delivery. Furthermore, the influence of size, crystal structures, and dispersion media (aqueous and biomimetic fluids) of MONPs on the magnetic properties, the degradation ability, and *T*_1_ and *T*_2_ NMR relaxivities (*r*_1_ and *r*_2_), has not been investigated yet, which is essential for designing efficient manganese oxide based theranostic NPs.

In this study, we investigated the effect of different manufacturing procedures on the chemical composition and size distribution of the MONPS. MONPs were synthesized in anoxic (N_2_) and normoxic (air) environments, with different metallic precursors (manganese oleate and manganese(ii) acetylacetonate). The effect of the size, chemical composition, and solvent on the degradation and corresponding magnetic and *T*_1_ and *T*_2_ NMR relaxivities was then assessed. The potential use of MONPs as a drug delivery vehicle was illustrated by surface functionalization of the NPs with l-3,4-dihydroxyphenylalanine (l-dopa) drug molecules, a precursor of neurotransmitters. These l-dopa functionalized MONPs may find useful applications in treating Parkinson's disease, which is related to dopamine deficiency.^35–37^l-dopa molecules offer functions beyond therapeutic effects. It has been known that l-dopa molecules have the ability to cross the blood–brain barrier (BBB) *via* the LAT1 transporter protein.^38^ The presence of hydrophilic groups in l-dopa molecules also makes MONPs hydrophilic and provides colloidal stability in the aqueous phase. This novel design of l-dopa functionalized MONPs will facilitate transport through the BBB. At the same time, the high NMR relaxivity enables *in vivo* tracking, and controlled release of l-dopa molecules to the target site (therapeutic effect).

## Results and discussion

**Fig. 1:**
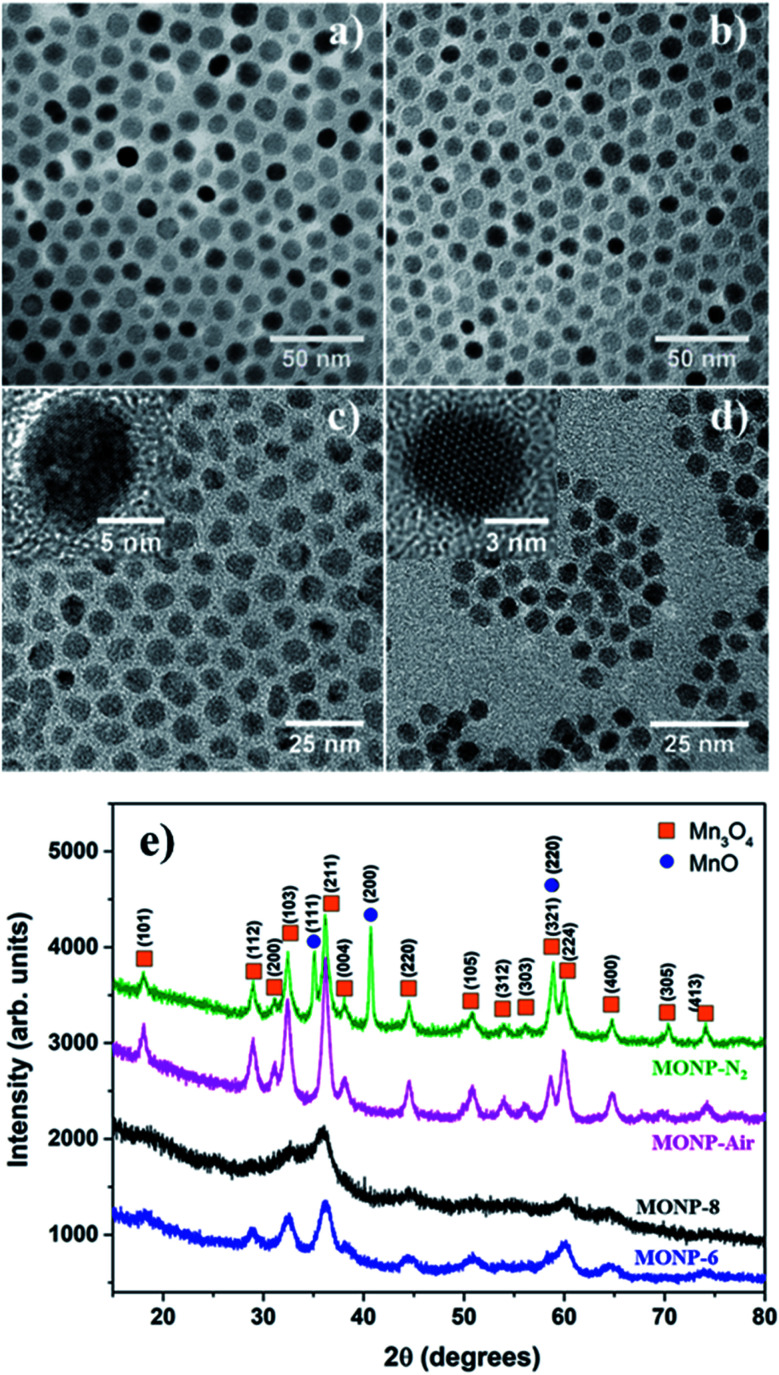
High resolution TEM images of MONPs synthesized by using different precursors and environments; (a) MONP-N_2_, (b) MONP-air, (c) MONP-8 (N_2_), and (d) MONP-6 (air). (e) XRD data indicate the presence of two phases (Mn_3_O_4_ and MnO).

The thermal decomposition approach was used to synthesize MONPs of different sizes and chemical compositions. The detailed synthetic protocol is described in the experimental section. Briefly, the procedure involved the gradual heating (5 °C min^−1^) of the reaction mixture containing the metal precursor (manganese(ii) acetylacetonate) and oleylamine to the reaction temperature (210 °C) in the presence of inert gas (N_2_) and air. Here, oleylamine acted as both solvent and a surfactant/capping molecule in this reaction. After the reaction, the mixture was cooled to room temperature and precipitated with toluene and acetone. Fig. 1a and b display the transmission electron microscopy (TEM) images of MONPs synthesized in the presence of N_2_ and air (hereafter referred to as MONP-N_2_ and MONP-air, respectively). The size of these NPs was measured to be 10 ± 2 nm (MNOP-N_2_) and 9 ± 2 nm (MONP-air) by counting more than 200 individual NPs (see Fig. S1a and b ESI†). To investigate the structural characteristics of NPs produced in different reaction environments, X-ray powder diffraction (XRD) was used. MONP-N_2_ showed distinct diffraction peaks at 18.04°, 28.96°, 31.17°, 32.42°, 36.20°, 38.10°, 44.49°, 50.80°, 59.98°, 64.76°, 70.37°, and 74.14° corresponding to tetragonal Mn_3_O_4_ (ICDD no. 04-008-0316), and 35.14°, 40.72°, and 58.85° corresponding to cubic MnO (ICDD no. 04-006-5363) as shown in Fig. 1e. Composition analysis using the ICDD PDF-4+ 2019 software revealed the presence of Mn_3_O_4_ (64%) and MnO (36%) phases in MONP-N_2_. MONP-air displayed diffraction peaks at 18.08°, 28.94°, 31.12°, 32.44°, 36.19°, 38.05°, 44.57°, 50.83°, 58.64°, 59.93°, and 64.76° corresponding to the Mn_3_O_4_ phase. The absence of additional diffraction peaks in MONP-air suggested the formation of NPs with the Mn_3_O_4_ phase only (*i.e.* the reaction in the presence of air facilitated the complete oxidation of NPs from MnO to Mn_3_O_4_). From these experiments it can be concluded that the reaction environment (N_2_*vs.* air) is an important factor that determines the chemical composition of MONPs but has little impact on the NP size.

To study the effect of precursor type on the size and chemical composition of MONPs, we replaced the manganese(ii) acetylacetonate precursor with manganese oleate. Thermal decomposition of manganese oleate in the presence of oleic acid and octadecene at 320 °C in N_2_ led to the formation of MONPs of 8 ± 1 nm (Fig. 1c and see Fig. S1c ESI†). Hereafter, these NPs will be referred to as MONP-8. The noticeable size difference between MONP-8 (∼8 nm) and MONP-N_2_ (∼10 nm), synthesised with difference precursors, can be explained by the chemical structure of the precursor molecule and its interaction with surfactant molecules. Manganese oleate decomposes slowly in the presence of oleic acid even at a higher temperature (320 °C) because of the strong bonding of three oleate molecules (fatty acids) to Mn ions and the strong interaction with the oleic acid solvent. In contrast, manganese acetylacetonate decomposes at a lower temperature (210 °C) due to the weak bonding of Mn ions to acetylacetonate ions as well as the weak interaction between the precursor and oleylamine. As a result, we obtained MONP-8 of a smaller size (prepared by manganese oleate) than MONP-N_2_ (prepared using manganese acetylacetonate).

When thermal decomposition of manganese oleate in the presence of oleic acid and hexadecene was carried out at 300 °C, MONPs of smaller sizes (6 ± 1 nm) were obtained (Fig. 1d and see Fig. S1d ESI†). Hereafter, these NPs will be referred to as MONP-6. This size reduction occurred by employing a lower boiling point solvent, hexadecene (285 °C), further suppressing the decomposition of the manganese oleate precursor. High resolution (HR) TEM images revealed the formation of single crystalline MONPs as displayed in the inset of Fig. 1c and d. The XRD pattern showed weak and broad diffraction peaks at 32.50°, 36.05°, 44.48°, and 60.06° for MONP-8, and relatively sharp peaks at 28.89°, 32.55°, 36.14°, 44.29°, 50.87°, 60.12°, and 64.37° for MONP-6. These diffraction peaks can be attributed to the presence of the Mn_3_O_4_ phase in both MONP-8 and MONP-6. This can be expected due to the complete oxidation of the NPs at a high reaction temperature (320 °C and 300 °C) in the oxygen-rich environment (oxygen source from the decomposition of both the manganese oleate precursor and oleic acid). Our synthesis results suggest that the size and chemical composition of MONPs can be varied by the appropriate selection of the metal precursor, solvent, and reaction environment.

**Fig. 2:**
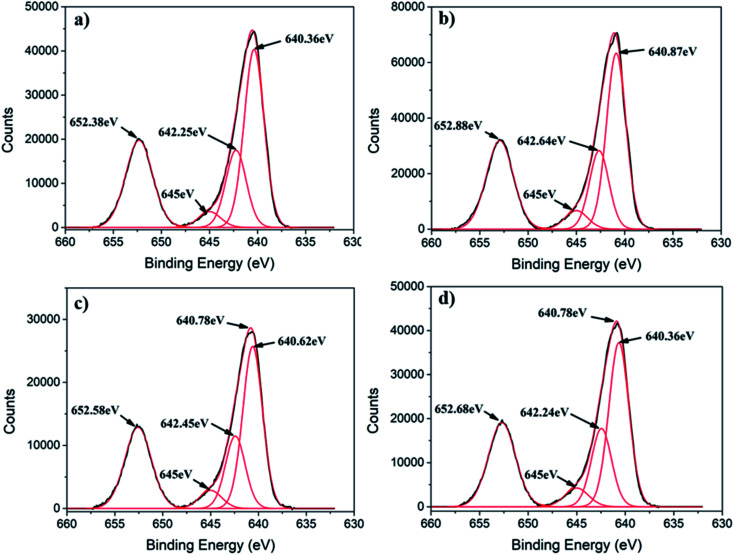
Deconvoluted high resolution Mn 2p XPS spectra of (a) MONP-N_2_, (b) MONP-air, (c) MONP-8, and (d) MONP-6.

Generally, Mn_3_O_4_ has a spinel structure in which O atoms are closely packed with Mn^2+^ in the tetrahedral sites and Mn^3+^ in the octahedral sites (Mn^2+^[Mn^3+^]_2_O_4_^2−^).^39^ Furthermore, Mn_3_O_4_ can exist in two possible oxide compositions, including MnO–Mn_2_O_3_ (with Mn^2+^ and Mn^3+^) and 2MnO–MnO_2_ (with Mn^2+^ and Mn^4+^).^39,40^ To determine the chemical state of Mn ions in different MONPs as synthesized in our work, X-ray photoelectron spectroscopy (XPS) was used to collect high resolution XPS spectra of Mn 2p. Due to the occurrence of spin–orbit coupling, Mn 2p spectra displayed a doublet separation of Mn 2p_3/2_ and Mn 2p_1/2_. A deconvoluted Mn 2p_3/2_ peak showed three distinct peaks. According to the reported literature, the deconvoluted Mn 2p_3/2_ peak indicates the presence of Mn^2+^ (640.10–641.12 eV) and Mn^4+^ (641.85–643 eV) species in all samples, including a satellite peak at 645 eV.^41^ For MONP-N_2_, the area ratio (*i.e.* molar ratio) of Mn^2+^ to Mn^4+^ is almost 2.5 : 1 (Fig. 2a). This agrees with the XRD data, showing the 64% Mn_3_O_4_ (2MnO–MnO_2_) phase and the remaining 36% MnO phase. For other samples (MONP-air, MONP-8, and MONP-6), the area ratio of Mn^2+^ to Mn^4+^ is almost 2 : 1, which agrees with theoretical oxide compositions (2MnO–MnO_2_), suggesting the presence of the Mn_3_O_4_ phase.

**Fig. 3:**
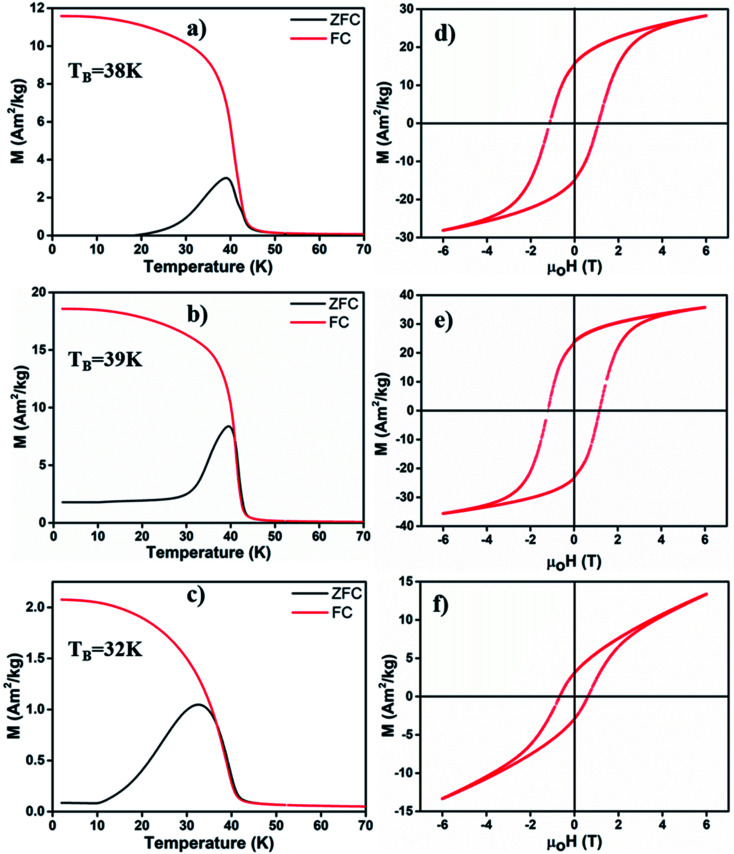
Zero field cooled (ZFC)-field cooled (FC) curves of (a) MONP-N_2_, (b) MONP-air and (c) MONP-8, recorded at an applied field of 60 kA m^−1^. Magnetic hysteresis loop (*M vs. μ*_0_*H*) of (d) MONP-N_2_, (e) MONP-air, and (f) MONP-8 measured at 5 K.

We employed a vibrating sample magnetometer (VSM) to determine the magnetic properties of different MONPs produced in this work. Bulk Mn_3_O_4_ is ferrimagnetic (FiM) with a Curie temperature (*T*_C_) of 42 K, and MnO is antiferromagnetic (AFM) with a bulk Néel temperature (*T*_N_) of 118 K.^42^ For magnetic property measurements, MONP-N_2_, MONP-air and MONP-8 were chosen for their dissimilar chemical composition (MONP-N_2_*vs.* MONP-air) and size (MONP-air *vs.* MONP-8). Fig. 3a–c show the temperature dependence of magnetization recorded by field cooled (FC) and zero field cooled (ZFC) procedures at weak magnetic fields of 40 kA m^−1^. For MONP-N_2_, and MONP-air, we observed a transition around 40 K (close to *T*_C_ of Mn_3_O_4_) in the FC curves, suggesting dominating magnetic behaviour by ferrimagnetic Mn_3_O_4_ (Fig. 3a and b). The peak in the ZFC curves indicates the blocking temperature (*T*_B_), which was observed to be ∼38 K and ∼39 K for MONP-N_2_ and MONP-air, respectively. These samples showed ferromagnetic behaviour at low temperatures (below 40 K) but were superparamagnetic at room temperature. MONP-8 exhibited a blocking temperature around 32 K (Fig. 3c), which is lower than that for MONP-N_2_ and MONP-air. This is consistent with the smaller size of the MONP-8 sample and the linear relationship between the nanoparticle volume (*V*) and blocking temperature (*T*_B_); *K*·*V* = 25*k*_B_*T*_B_ (where *K* is the anisotropy constant and *k*_B_ is Boltzmann's constant).^43^

To understand the magnetic behaviour of the synthesized MONPs, we measured the magnetic hysteresis curve at 5 K for an applied magnetic field ranging from −6 T to 6 T. A horizontal loop shift (*i.e.* the centre of hysteresis shifted towards the negative field) indicated the exchange bias effect in all samples (Fig. 3d–f). The exchange bias effect can originate from the coupling of spins from two different interfaces (FiM/AFM) or the coupling of ordered spins in the core with disordered spins in the outer surface.^44,45^ Here, the extent of exchange bias differed in all samples. The MONP-N_2_ sample displayed a significantly larger exchange bias field of ∼170 mT than MONP-air (∼47 mT) MONP-8 (∼81 mT). A large exchange bias field in MONP-N_2_ can be attributed to the presence of the AFM MnO phase (a larger contribution of FiM/AFM coupling) in MONP-N_2_. Inferring from the collective results from XRD, XPS, and magnetic properties, it is likely that thermal decomposition of manganese(ii) acetylacetonate in the presence of oleylamine and the N_2_ environment resulted in the formation of MONP-N_2_ with a core–shell (Mn_3_O_4_–MnO) structure. Comparing the exchange bias field of MONP-air and MONP-8 samples, MONP-8 showed a higher exchange bias field than MONP-air. XRD and XPS results suggest the formation of MONP-8 and MONP-air of the Mn_3_O_4_ phase. It thus rules out the possibility for an exchange bias effect due to the exchange coupling between AFM MnO (shell) and FiM Mn_3_O_4_ (core). In this case, the strong coupling between the disordered spins in the surface, and the ordered spins in the core of the NPs contribute to the exchange bias effect. Further, the exchange bias effect is strongly dependent on the size of the NPs.^46^ As the size of the NPs decreases, the fraction of disordered spins in the surface of NPs increases due to the larger surface area to volume ratio.^44,45^ The resultant effect is a stronger coupling between the disordered surface and ordered core spins with a decrease in size. Therefore, MONP-8 showed a large exchange bias effect in comparison to MONP-air. All samples displayed partial magnetic saturation behavior at high field strength (Fig. 3d–f). However, MONP-8 is less saturated than the others due to the strong surface effect (spin canting effect or high disordered spin structures) in small sized NPs. From the high resolution XPS spectra, XRD, and magnetic measurements, it can be concluded that the presence of oxygen during MONP synthesis, the metal precursor type, and solvent control the size and chemical composition (or the crystalline structure) of MONPs.

**Fig. 4:**
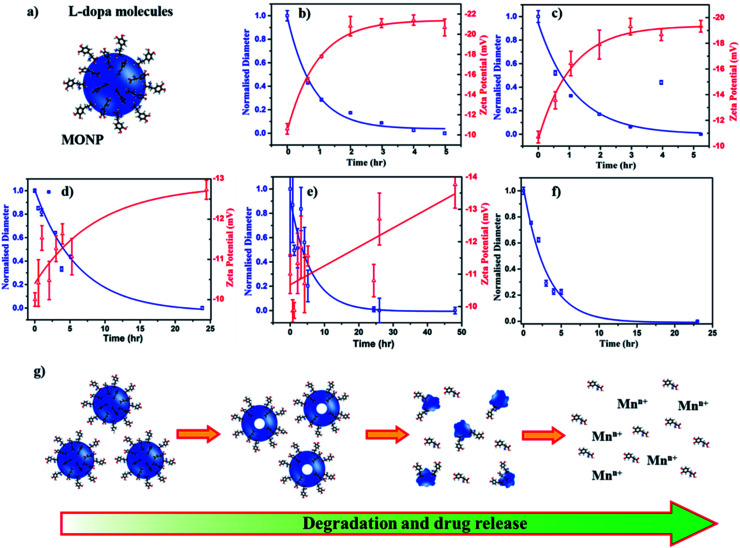
(a) Illustration of l-dopa functionalized MONP. Time-dependent degradation and zeta potential profiles of l-dopa functionalized (b) MONP-N_2_, (c) MONP-air, (d) MONP-8, and (e) MONP-6 particles in deionized water. (f) Time-dependent degradation of l-dopa functionalized MONP-N_2_ in biological mimicking fluid (25 v/v% FBS in deionized water). (g) Schematic illustration of MONP degradation in aqueous solution and release of drug molecules.

To assess the potential of MONPs as drug delivery vehicles, we functionalized MONP-N_2,_ MONP-air, MONP-8, and MONP-6 with l-dopa molecules (Fig. 4a). Conjugation of l-dopa molecules to these NPs provides stability in a polar biological environment and can also be used as a drug for Parkinson's diseases. To confirm the successful functionalization of MONPs with l-dopa molecules, we collected XPS survey spectra and determined at% of elements C, N, O, and Mn (see Table S1, ESI†). For oleylamine coated MONP-N_2_ and MONP-air, an increase in the N content can be noticed. Oleic acid capped MONP-8 and MONP-6 displayed the N content after surface functionalization. The presence of the N signature in the survey and high resolution XPS N 1s spectra of MONP-6 is shown in Fig. S2 (see the ESI†). The three distinct peaks at 285 eV (C–C), 286.5 eV (C–N/C–O), and 288.2 eV (O–C=O) in the deconvoluted C 1s spectra further verified the presence of l-dopa molecules on the MONP-6 surface (Fig. S3, see the ESI†). Once these MONPs are dispersed into an aqueous phase, they are prone to degradation followed by the release of surface drug molecules. Degradation kinetics of NPs is one of the important factors for designing MONP based drug vehicles because it is strongly related to the release kinetics of drug molecules from the NP. To investigate the degradation rate of l-dopa functionalized MONP-N_2_, MONP-air, MONP-8, and MONP-6 in the aqueous phase, the hydrodynamic diameter and zeta potential of these NPs were monitored over time using the dynamic light scattering (DLS) technique (Fig. 4b and e and S4, ESI†). For quantitative comparison between the degradation rate of various l-dopa functionalized NPs, an exponential fit was applied on the normalized size of the NP as a function of time:

1 *D*_h_ = Δ*D*e^−*xt*/*τ*^ + *D*_h,∞_

where *D*_h_ = hydrodynamic diameter, *t* = time after dispersion (hours), *τ* = time constant, Δ*D* is the maximum change in the hydrodynamic diameter and *D*_h,∞_ is the hydrodynamic diameter reached asymptotically after a long degradation time.

Fitting of the degradation curve with eqn (1) gives a time constant *τ*, which follows the following trend for l-dopa coated MONPs: 0.91 hours (MONP-N_2_) < 0.96 hours (MONP-air) < 5.87 hours (MONP-8) < 6.15 hours (MONP-6). In all samples, a large proportion of MONPs degraded within the first 5 hours, with a significant decrease in the hydrodynamic diameter in the first hour. The degradation of MONPs in the aqueous solution can be explained based on the nanoscale Kirkendall effect (a process of atomic diffusion originated by an exchange of vacancies instead of the interchange of atoms) as discussed in our earlier work.^28^ Whilst Mn atoms diffuse outward and get oxidized at the surface, there is an influx of vacancies towards the centre. As a result, hollow MONPs are formed (Fig. 4g). Further oxidation of the shell leads to a porous shell, which breaks into small fragments. With time, the process of oxidation, porous shell formation, and shell breaking continues until the MONPs are completely dissolved in the aqueous phase. Comparing the degradation of l-dopa functionalized MONP-N_2_ and MONP-air samples, the faster degradation of MONP-N_2_ than MONP-air can be attributed to the difference in their chemical composition. Our results reveal the overall decrease in the degradation rate along with the increase in the size of l-dopa functionalized MONP. As the size of the NPs decreases, smaller NPs get oxidized readily compared to large NPs due to their large surface-to-volume ratio.^47^ The degradation products formed (due to the oxidation) within smaller NPs diffuse rapidly to the surface because of the shorted diffusion distance. In contrast, the degradation products formed in the large NPs diffuse slowly to the outer surface (a longer diffusion path), during which the degradation of remaining materials can occur. This is consistent with the size-dependent degradation of polymeric NPs.^48^ Therefore, the degradation of rate increases with the decrease in the size of the NP. From Fig. 4b–e, it can be noticed that the zeta potential of l-dopa coated MONPs increases (more negative) with the increase in the degradation time. l-dopa molecules are anchored to the surface of MONPs *via* two hydroxyls present in the molecules. Initially, l-dopa coated MONPs display a low negative surface charge due to the presence of amine and carboxyl groups on their surface. As the degradation of NPs proceeds, the increase in the negative surface charge is expected because of the release of l-dopa molecules (*i.e.* the increased proportion of hydroxyl group exposed to the aqueous solution).

The results from the degradation study also reveal the importance of the metallic precursor (manganese acetylacetonate *vs.* manganese oleate), reaction environment (N_2_*vs.* air) and solvent (oleylamine and octadecene *vs.* hexadecene). These reaction parameters control the degradation rate of l-dopa coated MONPs, which can be tuned based on time (from hours to days) depending on the synthetic parameters used for the fabrication of MONPs. An efficient drug delivery vehicle must transport the drug and release the drug in a controlled manner. In traditional drug delivery systems based on inorganic nanoparticles, the drug molecules are usually attached to NPs with covalent bonds. Breaking these covalent bonds in an *in vivo* environment can be challenging. The unreliability of breaking covalent bonds may impair the efficiency of drug delivery. Our l-dopa functionalized MONP based drug delivery system eliminates these problems, thus providing the release of l-dopa molecules from the MONPs as they disintegrate in an aqueous solution. Furthermore, the release of drugs can be controlled by selecting the appropriate MONP type (MONP-air or MONP-N_2_) and size. It should be noted that the degradation ability and subsequently, the release of drug molecules can be different when the MONPs are introduced into a living system. This is because blood proteins and biomolecules immediately compete to adhere to the MONP surface, forming a protein corona, with the composition dependent on the NP surface chemistry and the physical characteristics.^49^ The biological identity, controlled by surface biomolecules, can promote varying bio-interactions and cell functionality such as increased cellular uptake, proliferation or modulating the immune responses.^50^ To observe the degradation of MONPs in biological fluids (mimicking *in vivo* microenvironment), we dispersed l-dopa functionalized MONP-N_2_ in a protein-rich biological fluid containing bovine serum (25 v/v% fetal bovine serum in deionized water). DLS was used to measure the influence of the biological fluids on the degradation of MONPs. The degradation profile was generated by changes in the hydrodynamic radius and fitting of an exponential decay model. The time constant for degradation was measured to be 3.05 hours, which is higher than 0.92 hours for the MONP-N_2_ sample dispersed in deionized water (Fig. 4f). There was a significant change in the degradation of the MONP-N_2_ sample when dispersed in a biological fluid. We did not notice any sedimentation of MONPs on visual inspection due to aggregation in the biological fluid during the time-dependent degradation study. This indicates the stability of MONPs in a biological fluid. In water, l-dopa functionalized MONPs oxidized rapidly due to easy accessibility of water molecules at the surface of NPs. On introducing l-dopa functionalized MONPs to the biological fluid containing plasma proteins, we suspected the formation of a thick layer of protein corona around the MONPs. The protein corona formation led to low accessibility of water molecules at the surface of MONPs.^51^ As a result, the sluggish oxidation caused the low degradation rate of MONPs in the biological fluid. Therefore, the degradation rate of MONPs, and subsequently, the release of drug molecules from the surface can be different in an *in vivo* environment.

**Fig. 5:**
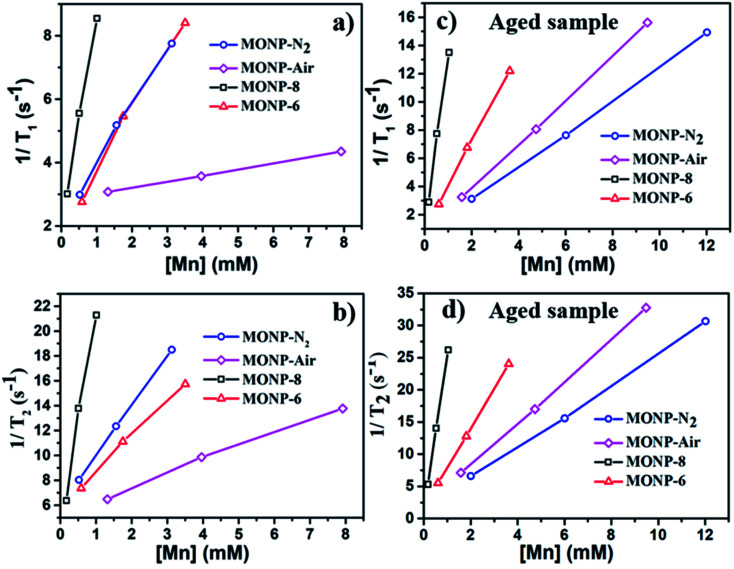
*T*_1_ and *T*_2_ relaxation rates as a function of Mn concentration determined for l-dopa functionalized MONP-N_2_, MONP-air, MONP-8, and MONP-6 particles (a and b) immediately after dispersion in deionized water and (c and d) 24 hours aging in deionized water.

MONPs can also be used as MRI contrast agents. The NMR relaxivities (*r*_1_ and *r*_2_) of the MONPs were measured in 0.5 T time-domain relaxometry (Bruker, Minispec mq20) (Fig. 5 and Table 1). All measurements were conducted at 40 °C. The NMR relaxivities of the MONP solutions were measured in water within 1 hour and 24 hours after dispersion. MONP-N_2_ showed higher longitudinal (*r*_1_ = 1.8 mM^−1^ s^−1^) and transverse (*r*_2_ = 4.0 mM^−1^ s^−1^) relaxivities compared to MONP-air (where *r*_1_ = 0.2 mM^−1^ s^−1^ and *r*_2_ = 1.1 mM^−1^ s^−1^) (Fig. 5a and b). This difference in the relaxivities of MONP-N_2_ and MONP-air cannot be simply associated with their size (10 ± 2 nm and 9 ± 2 nm respectively) and chemical composition because both the size and chemical composition change with time due to the degradation of MONPs after their dispersion in deionized water. The results from the degradation study revealed a higher rate of degradation of MONP-N_2_ than of MONP-air. A rapid decrease in the size of MONP-N_2_ along with time can also increase the proportion of the magnetically disordered spin structure (canted surface spins) due to the increasing surface-to-volume ratio.^52^ At the same, a change in the chemical composition due to the degradation of l-dopa coated MONP-N_2_ will also lead to the increased proportion of Mn^2+^ ions (a high number of unpaired electrons in the 3d configuration of Mn^2+^) in the solution compared to MONP-air. To verify this, we collected high resolution XPS spectra of Mn 2p, and determined the relative intensity ratio of deconvoluted peaks corresponding to Mn^2+^ and Mn^4+^ (see Fig. S5 and S6, ESI†). The spectra were collected after the dispersion of l-dopa coated MONPs in the aqueous phase, followed by immediate drying prior to XPS. The results showed a relatively higher proportion of Mn^2+^ ions in MONP-N_2_ compared to MONP-air, *i.e.*, a higher ratio of Mn^2+^ to Mn^4+^ in the MONP-N_2_ (∼2.6) sample than in MONP-air (∼1.8). Therefore, both the increase in the magnetically disordered structure and improved accessibility of water molecules to excess Mn^2+^ ions are expected to contribute to higher *T*_1_ and *T*_2_ relaxivities of MONP-N_2_. When MONP-N_2_ and MONP-air were aged for 24 hours under similar conditions, both the samples displayed similar *r*_1_ values (*r*_1_ = 1.2 mM^−1^ s^−1^) (Fig. 5c and d, and Table 1) because both the aged samples showed a similar Mn^2+^ to Mn^4+^ ratio (∼1.7). Our results indicate the importance of considering degradation in the evaluation of the induced MRI contrast of MONPs, which is also dependent on the fabrication method.

**Table 1:** Summary of NMR relaxivities of freshly dispersed and 24-hour aged (highlighted in blue bracket) l-dopa functionalized MONPs in deionized water

Sample	Chemical composition	Size (nm)	*r*_1_ (mM^−1^ s^−1^)	*r*_2_ (mM^−1^ s^−1^)	*r*_2_/*r*_1_
MONP-N_2_	Mn_3_O_4_–MnO	10 ± 2	1.8 (1.2)	4.0 (2.4)	2.2 (2.0)
MONP-air	Mn_3_O_4_	9 ± 2	0.2 (1.2)	1.1 (3.3)	5.5 (2.8)
MONP-8	Mn_3_O_4_	8 ± 1	6.5 (12.2)	17.4 (24.1)	2.7 (2.0)
MONP-6	Mn_3_O_4_	6 ± 1	1.9 (3.1)	2.9 (6.1)	1.5 (2.0)

Next, we compared the relaxivities of l-dopa coated MONP-8 and MONP-6 samples of similar chemical compositions fabricated by a similar protocol, but they differ in their sizes. Moreover, both the samples slowly degrade in the aqueous phase. The MONP-8 sample displayed increased longitudinal (*r*_1_ ∼ 6.5 mM^−1^ s^−1^) and transverse (*r*_2_ ∼ 17.4 mM^−1^ s^−1^) relaxivities in comparison to MONP-6 (*r*_1_ ∼ 1.9 mM^−1^ s^−1^ and *r*_2_ ∼ 2.9 mM^−1^ s^−1^) as shown in Fig. 5a, b and Table 1. The large value of *r*_2_ for the MONP-8 sample is expected, which results from a large magnetization value with the increased NP size.^53^ The longitudinal relaxivity (*r*_1_) can be correlated with the size based on the number of water molecules in close proximity to the NP surface, as well as, with the molecular mobility of the NP.^53^ A decrease in the size of NPs will allow a smaller number of Mn atoms to be exposed to water molecules and, thus, a low *r*_1_ value for MONP-6. Based on high resolution XPS spectra, the large *r*_1_ value can also be explained based on the high Mn^2+^ to Mn^4+^ ratio for MONP-8 (∼1.9) compared to MONP-6 (∼1.8) because of a relatively faster degradation rate of MONP-8. Therefore, MONP-8 shows high longitudinal and transverse relaxivities compared to MONP-6. A similar trend can also be seen for 24 hours aged MONP-8 and MONP-6 samples (Fig. 5c, d and Table 1). However, it can be noticed that large size MONP-air (9 ± 2 nm) of the Mn_3_O_4_ phase displays low *r*_1_ and *r*_2_ values compared to MONP-8 and MONP-6. This difference can arise from the different chemical composition and degradation rate of MONP-air because these NPs were produced by a different protocol.

**Fig. 6:**
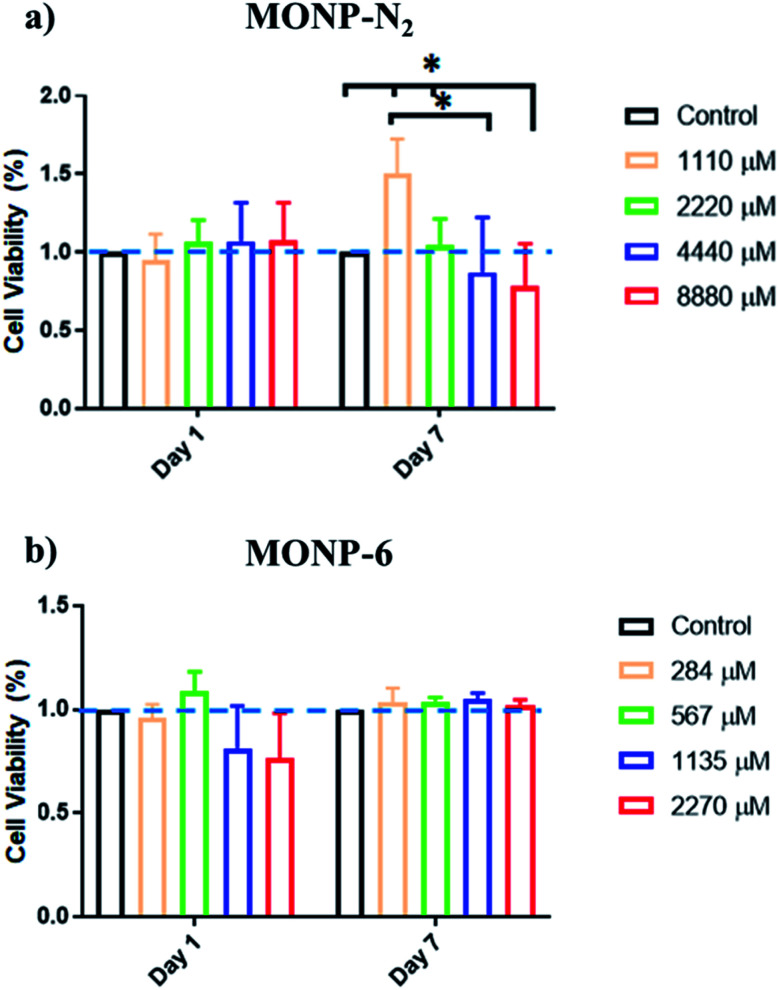
Cell viability of human foreskin fibroblasts incubated with l-dopa coated (a) MONP-N_2_ and (b) MONP-6 at various concentrations (0–8880 μM Mn), as determined by MTS assay. All data have been normalised to the control.

The viability of human foreskin fibroblast cells following co-incubation with l-dopa functionalized MONPs was evaluated using the MTS assay. We chose two different NPs (MONP-N_2_ and MONP-6) with different sizes and chemical compositions for the cell study. Our results showed that fibroblast viability was not significantly affected when these cells were cultured with MONP-N_2_ up to 2200 μM Mn for 7 days. However, at higher concentrations (4440 and 8880 μM, a significant downregulation in fibroblast viability compared to the control and/or to the low dose (1100 μM groups (Fig. 6a)) can be noticed. In contrast, when cultured with MONP-6 at a dose between 284 and 2270 μM NPs for 7 days, fibroblast viability remained constant and did not change (Fig. 6b). The difference in fibroblast cell viability for two different samples (MONP-N_2_ and MONP-6) could be associated with the difference in their chemical composition as well as the size. Because toxicity is only observed at a higher dose of MONP-N_2_, the concentration itself may be a participating factor. As there could be multiple contributing factors, further studies are required to investigate the root cause of the biocompatibility differences.

## Conclusions

In summary, we demonstrated the influence of the metal precursor type and reaction environment (N_2_ and air) on the size, chemical composition, and physicochemical properties (magnetic properties, degradation rates, and NMR relaxivities) of MONPs. Thermal decomposition of manganese(ii) acetylacetonate in the presence of N_2_ and air led to the formation of MONP-N_2_ (consisting of Mn_3_O_4_ and MnO phases) and MONP-air (single phase Mn_3_O_4_). MONPs of smaller sizes (MONP-8 and MONP-6) and single phase Mn_3_O_4_ can be synthesized by using a manganese oleate precursor. Our results indicated that the degradation rate and the release of l-dopa drug molecules depend on the chemical composition and size of MONPs. MONP-N_2_ and MONP-air degraded significantly faster than MONP-8 and MONP-6, suggesting the release of l-dopa molecules from the MONP surface. Furthermore, MONP-N_2_ degraded slowly in biological media compared to their degradation in the aqueous phase which has been attributed to the formation of a protein corona around NPs that may have shielded to prevent the rapid oxidation of MONP-N_2_. We also show that the NMR relaxivities of l-dopa functionalized MONPs of different chemical compositions and sizes differ. This is due to the change in physiochemical properties (size and composition) as NPs degrade in aqueous solution. Finally, our preliminary assessments of a fibroblast cell viability study showed non-cytotoxicity, which could depend on the size and composition of the NPs. Overall, this work opens a new avenue toward designing simple self-degradable MONPs armed with multiple capabilities (targeting, diagnosis, and therapy).

## Conflicts of interest

There are no conflicts to declare.

## Supplementary Material

NA-003-D0NA00991A-s001
