# Supplementary material for: Tuneable manganese oxide nanoparticle based theranostic agents for potential diagnosis and drug delivery
Source: Nanoscale Adv. 2021 Jun 7;3(14):4052–61. doi: 10.1039/d0na00991a (PMC9419237; doi:10.1039/d0na00991a)
Supplement: NA-003-D0NA00991A-s001 [file NA-003-D0NA00991A-s001.pdf]

Electronic Supplementary Material (ESI) for Nanoscale Advances.
This journal is © The Royal Society of Chemistry 2021

# Supporting Information

## Tuneable manganese oxide nanoparticle based theranostic agent for potential diagnosis and drug delivery

Kingsley Poon[a], Zufu Lu[a], Yves De Deene[b], Yogambha Ramaswamy[a], Hala Zreiqat*[a] and Gurvinder Singh*[a]

*[a]ARC Centre for Innovative BioEngineering, Tissue Engineering and Biomaterials Research Unit, Tissue Engineering and Biomaterials Research Unit, Sydney Nano Institute, School of Biomedical Engineering, The University of Sydney, NSW 2008, Australia.*

*[b]The Biomedical Engineering Laboratory, Department of Engineering, Macquarie University, Sydney, 2109, Australia.*

***Email:*** *hala.zreiqat@sydney.edu.au, gurvinder.singh@sydney.edu.au*

## Experimental method

**Materials:** Manganese (II) acetylacetonate, oleic acid (technical grade, 90%), 1-octadecene (technical grade, 90%), 1-hexadecene (≥98.5%), chloroform (anhydrous, ≥99%), dimethyl sulfoxide (anhydrous, ≥99%), oleylamine (technical grade, 70%), 3,4-dihydroxy-L-phenylalanine (L-dopa, ≥98%) and manganese(II) chloride (≥98%) were purchased from Sigma Aldrich. Sodium oleate (97%) was received from TCI, Europe.

**Synthesis of MONP-$N_2$ and MONP-Air:** Manganese oxide nanoparticles (MONPs) were synthesized by thermal decomposition. To synthesize MONP-$N_2$, 1 g of manganese (II) acetylacetonate ($Mn(acac)_2$) was dissolved in 20mL oleylamine in a three-neck round bottom flask (100mL) equipped with a condenser. With vigorous stirring and under a nitrogen environment, the temperature of the reaction mixture was slowly raised to 210°C with a temperature increase of 5°C $min^{-1}$ and kept at 210°C for 5 hours. The mixture was then allowed to cool down to room temperature before precipitation using 20 mL of toluene and 60 mL of acetone. Following centrifugation, the supernatant was discarded, and the nanoparticles (NPs) were washed with toluene and acetone. The NPs were then dispersed into toluene. The similar reaction was performed under air to obtain MONP-Air.

**Synthesis of MONP-8 and MONP-6:** These NPs were synthesized by thermal decomposition of manganese oleate. Firstly, the manganese oleate precursor was synthesized by mixing manganese (II) chloride hexahydrate (20 mmol), sodium oleate (60 mmol), deionized water (30 mL), hexane (70 mL) and ethanol (40 mL) in a 250 mL round bottom flask. The reaction mixture was vigorously stirred at 70 °C for 4 h under argon atmosphere. After the reaction, the organic component was separated from the aqueous phase and washed three times with deionized water to remove reaction byproducts. The product was further purified in a rotary evaporator to remove residual hexane, ethanol and water, before being transferred to a glass vial and stored in a fridge (4 °C). For the synthesis of MONP-8, the reaction mixture containing 3.4 g of manganese oleate, 1.2 mL oleic acid, and 50 mL of 1-octadecene (1-hexadecene for MONP-6) was heated to an end temperature of 320°C (300°C for MONP-6) at the rate of 3°C $min^{-1}$, and kept for 45 minutes at the end temperature. After the reaction, MONPs were obtained by precipitating the reaction mixture with 40 mL toluene and 120 mL acetone.

**Functionalization of MONPs with L-dopa:** 5 mg of dried MONPs was dispersed in 5 mL chloroform and mixed with 20 mg of L-dopa and 3 mL DMSO. The mixture was heated to 70°C and left to react for 1 hour under constant stirring. The surface-coated nanoparticles were separated by centrifugation (4400 rpm, 10 minutes). Following the removal of the supernatant, the precipitate was dispersed in acetone and centrifuged again to remove the unbound L-dopa molecules. The functional NPs were dispersed in deionized water prior to use.

**Characterization:** Transmission electron microscopy (TEM) images were taken with a TEM (Philips CM30), operating at 120 kV and JEOL 2100 operating at 200 kV. X-ray diffraction (XRD) analysis was performed with an X-ray diffractometer (Malvern PANanalytical X'pert Pro) using Cu Kα ($\lambda$ = 0.154nm) in the 2θ range of 15° – 80°. Spectral data was analysed using ICDD PDF-4+ 2019 software. X-ray photoelectron spectroscopy (XPS) measurements on MONPs were performed using XPS K-Alpha spectrometer (Thermo Fischer Scientific) with a monochromatic AL K-Alpha (1486.6eV) X-ray source. Survey spectral peaks were obtained using 1eV energy step and pass energy of 200eV. The high resolution XPS spectra were collected using 0.1eV energy steps and pass energy of 50 eV. XPS data was analysed using

Thermo Avantage V5.9902 whilst elemental spectral data was analysed using CasaXPS. Background subtraction of the elemental spectra was performed using the Shirley method and peak deconvolution with a Gaussian-Lorentzian function (70% Gaussian, 30% Lorentz).

**Time dependent degradation study:** To measure the degradation rate of MONPs in water, 5 mg functionalised nanoparticles were dispersed in 5 mL of MilliQ water and sonicated to ensure proper mixing. The hydrodynamic radius measurements, performed using a Malvern Zetasizer Nano ZS instrument at 25°, were taken at 0, 0.5, 1, 2, 4, and 24 hours after dispersion. Prior to each measurement, the solution was sonicated for 2 minutes to ensure proper dispersion. To measure the degradation under physiological conditions, the protocol above was modified. 5 mg of functionalised nanoparticles was dispersed in 5 mL of physiological fluid (25 v/v% FBS in MilliQ water). Prior to each measurement, the nanoparticles were diluted with MilliQ water (6.25 v/v% nanoparticle solution). All results were analysed using the Zetasizer software.

**Relaxivity measurement:** A 0.5 T time domain NMR relaxometry (Minispec Analyzer MQ20, Bruker) was used for $T_1$ and $T_2$ measurements. A total of 800 μL of each sample was poured in a 10 mm sample tube. All the measurements were made at 40°C using a thermostatic circulator water bath. $T_2$ decay curves were acquired using a Carr-Purcell-Meiboom-Gill (CPMG) pulse sequence with a repetition time (TR) of 1s and 1000 spin-echoes with an echo time spacing of 0.25 ms. $T_1$ values were obtained using an inversion-recovery pulse sequence with a repetition time (TR) of 2 seconds and 20 logarithmically spaced inversion times ranging from 0.5 ms to 1 s. Relaxivities ($mM^{-1}s^{-1}$) were calculated by taking the inverse of $T_1$ and $T_2$ times (seconds) and dividing these values by the total metal ion (Mn) concentration (mM) for each sample as determined by inductively coupled plasma mass spectroscopy (ICP-MS).

**Magnetic properties:** Magnetic properties of MONPs were measured with a quantum design physical properties measurement system (PPMS) with vibrating sample magnetometer (VSM). ZFC/FC curves. M(H) were measured by cooling the sample at zero field from room temperature to 2K and warming up from 2K to 380K at the rate of 1K/min with small field 500 Oe. M(H) curves at 5K were measured from sweeping the field from -60kOe to +60kOe.

**Cytotoxicity:** Human foreskin fibroblasts were cultured in complete media containing DMEM, supplemented with 10% (v/v) fetal calf serum, 30 mg/ml penicillin, and 100 mg/mL streptomycin. Cells were cultured at 37°C with 5% $CO_2$, and culture medium changed every 3 days until cells were passaged at 80-90% confluence. All fibroblasts were at passage 12. Cells were then seeded at a density of $10x10^3$ cells/well before incubation with MONPs at various concentrations. The cell viability was assessed after 1 day, 3 days and 7 days with an MTS assay (using Cell Titre 96 Aqueous non radiologic cell proliferation assay kit (Promega, USA)) following the manufacturer's instructions. The absorbance was recorded using a Multiskan Go Spectrophotometer at a wavelength of 490nm. ICP-MS was used to quantify the concentration of MONPs present in each group.

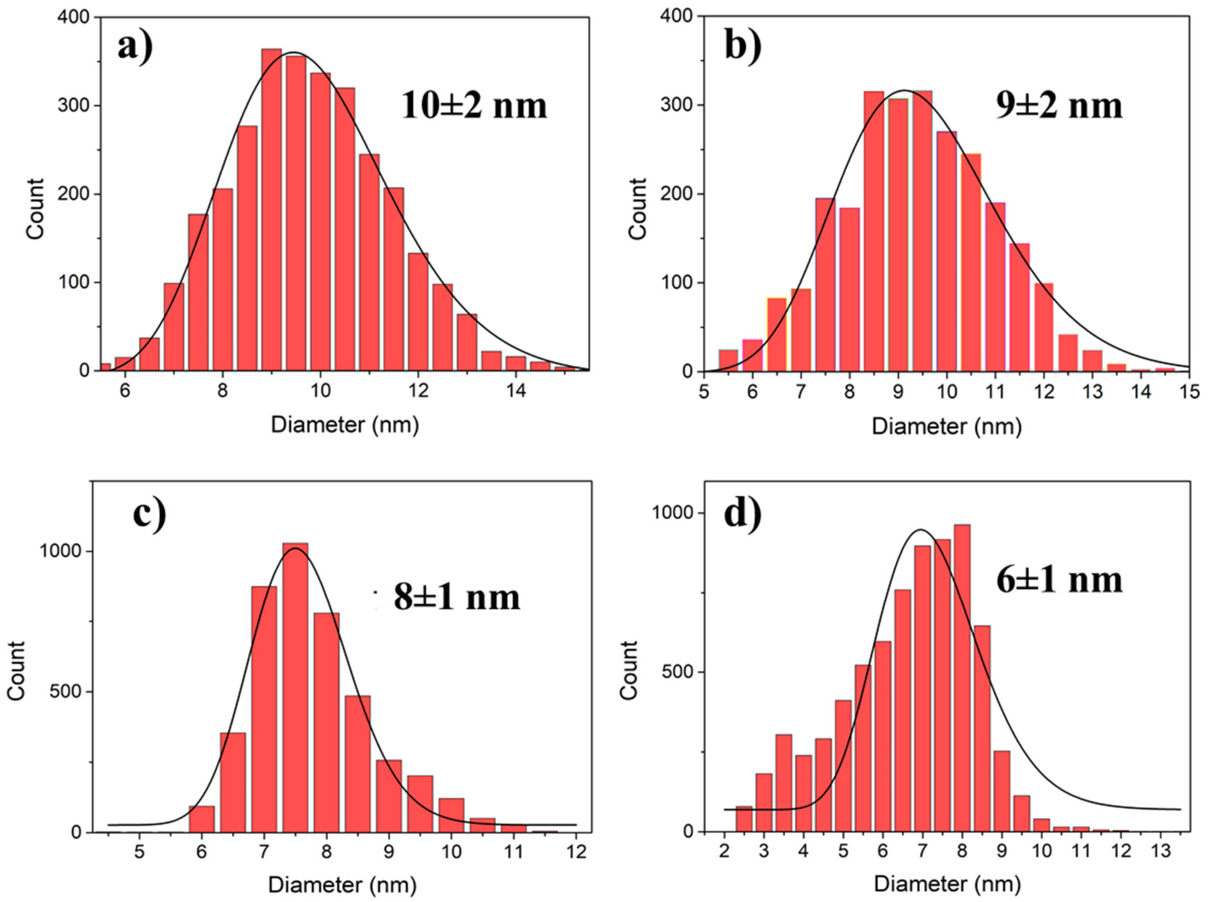


**Fig. S1** Histograms display size distribution of MONPs determined from TEM images; a) MONP-$N_2$, b) MONP-Air, c) MONP-8 and d) MONP-6.

**Table S1.** Elemental (at%) of MONPs determined from XPS survey spectra before and after the surface functionalization of MONP with L-dopa. Oleylamine capped MONP-$N_2$ and MONP-Air were synthesized in the presence of oleylamine solvent. Oleic acid capped MONP-8 and MONP-6 were synthesized in the presence of octadecene and oleic acid.

| Sample Id | C (at%) | O (at%) | N (at%) | Mn (at%) |
|---|---|---|---|---|
| MONP-$N_2$ | 68.4 | 20.2 | 1.9 | 9.5 |
| L-dopa MONP-$N_2$ | 69.1 | 21.0 | 4.2 | 5.7 |
| MONP-Air | 53.5 | 30.4 | 0.9 | 15.2 |
| L-dopa MONP-Air | 61.3 | 29.7 | 0.7 | 8.2 |
| MONP-8 | 78.5 | 15.0 | - | 6.5 |
| L-dopa MONP-8 | 74.3 | 17.8 | 4.5 | 3.4 |
| MONP-6 | 69.8 | 21.1 | - | 9.1 |
| L-dopa MONP-6 | 70.6 | 20.9 | 4.7 | 3.8 |

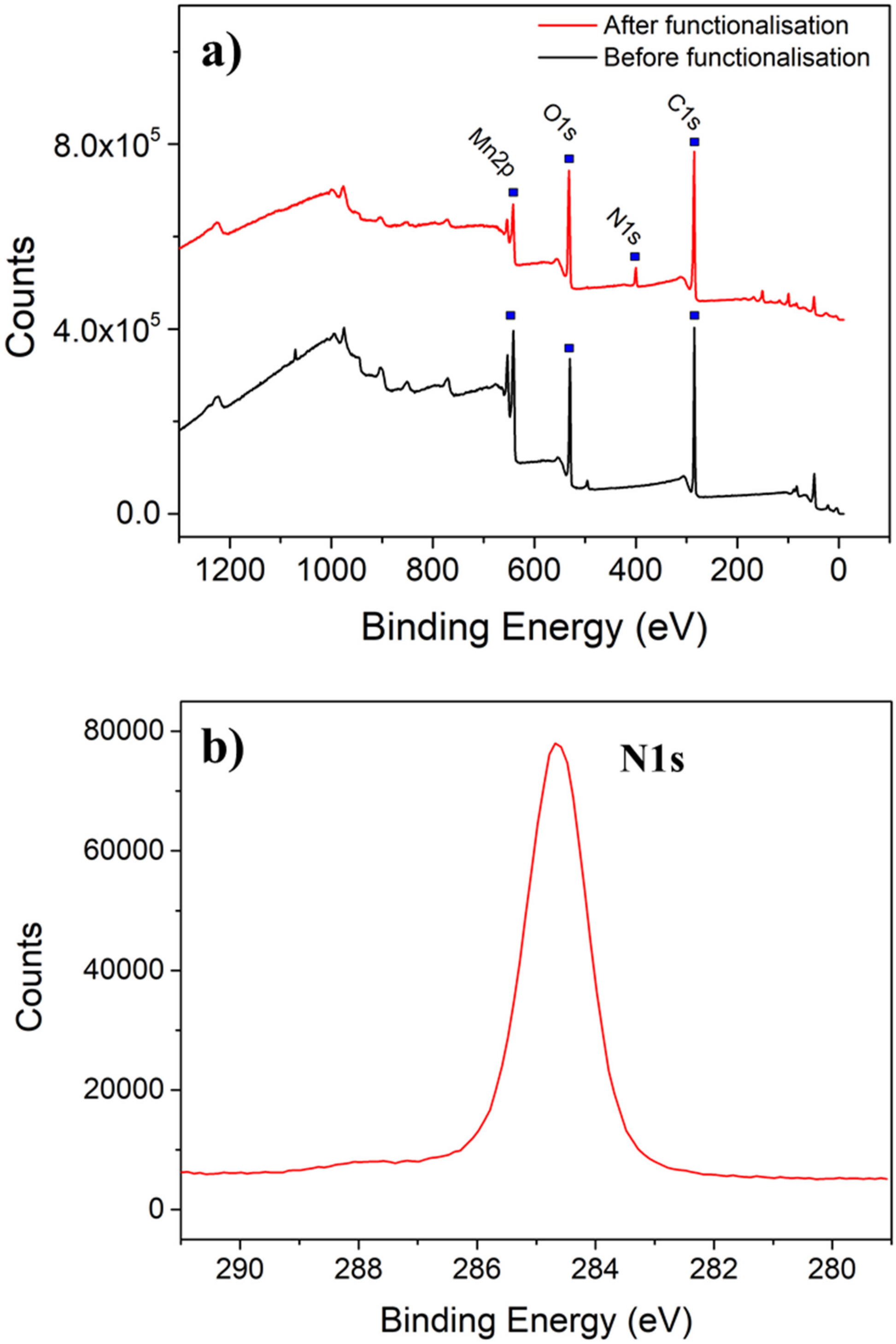


**Fig. S2** a) XPS survey scans of MONP-6 before and after the functionalization with L-dopa molecules. b) High resolution XPS spectrum of N1s after the functionalization of MONP-6.

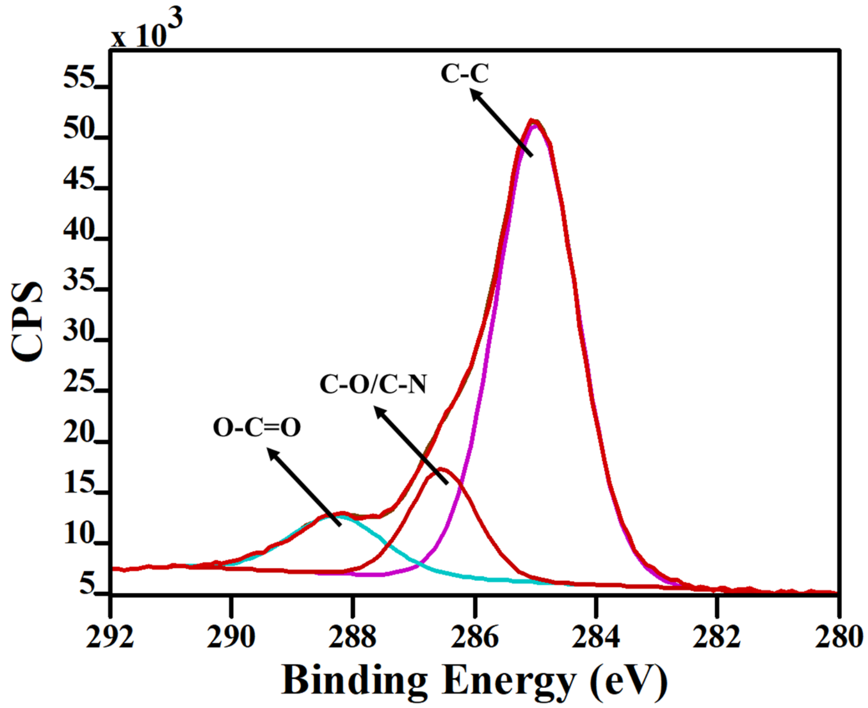


**Fig. S3** High resolution XPS spectrum of C1s (MONP-6).

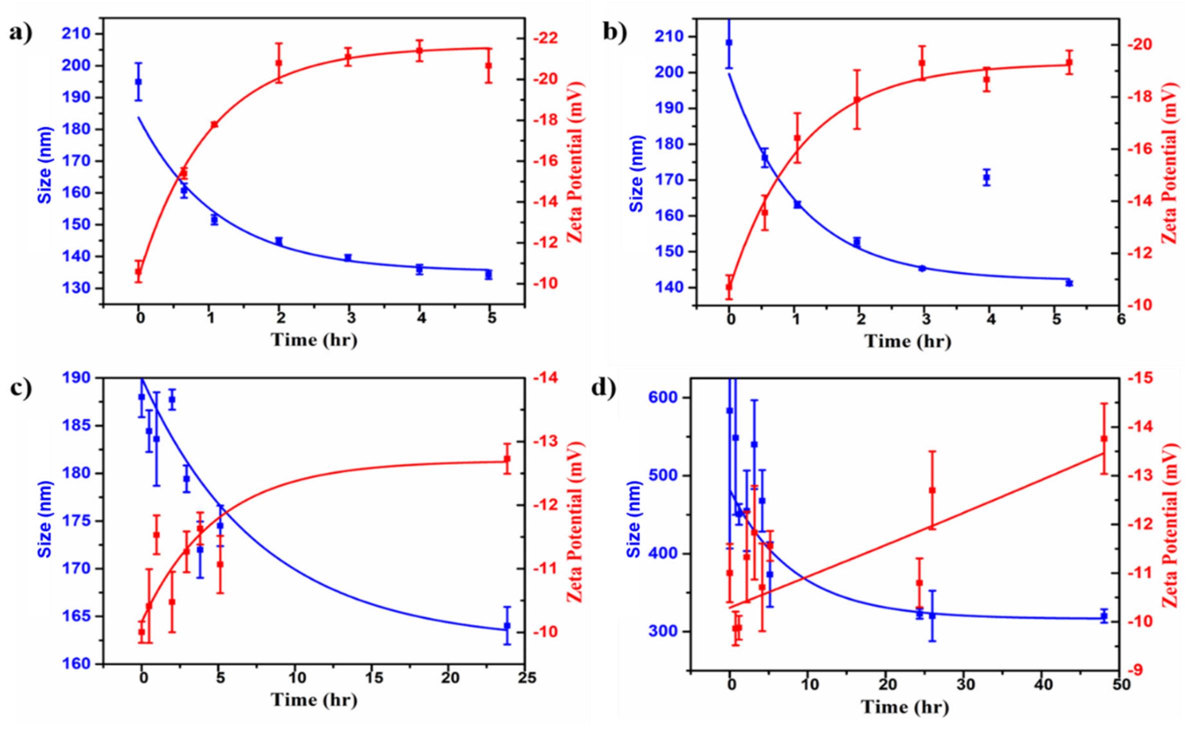


**Fig. S4** Time-dependent degradation and zeta potential profiles of L-dopa functionalized a) MONP-$N_2$, b) MONP-Air, c) MONP-8, and d) MONP-6 particles in deionized water.

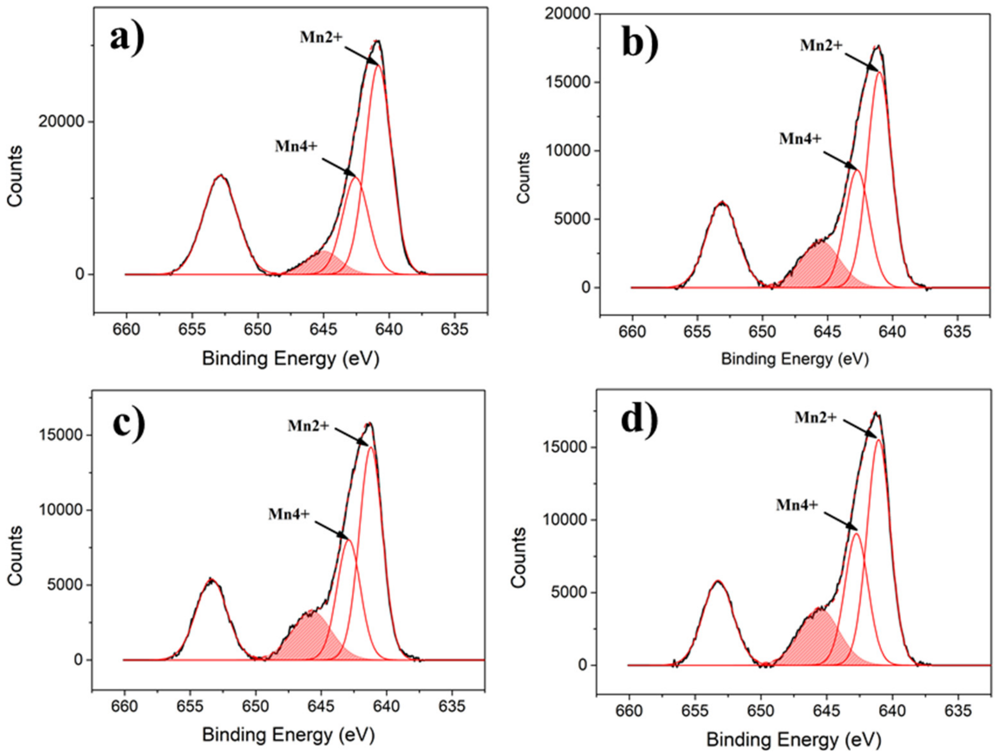


**Fig. S5** High resolution XPS spectra of Mn2p obtained after the dispersion of L-dopa functionalized MONP-$N_2$ particles in deionised water for different time, a) 0 hr, b) 1 hr, c) 4 hr and d) 24 hr.

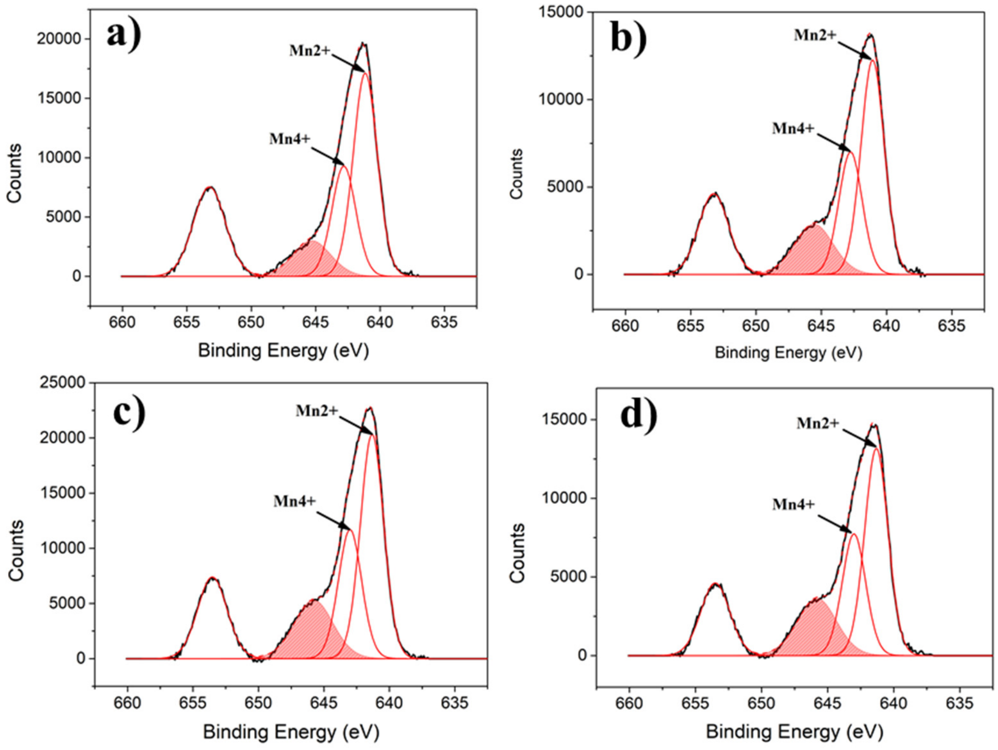


**Fig. S6** High resolution Mn2p spectra after the dispersion of L-dopa coated MONP-Air particles in water for different time, e) 0 hr, f) 1 hr, g) 4 hr and h) 24 hr.
